# Prolonged high-fat diet induces gradual and fat depot-specific DNA methylation changes in adult mice

**DOI:** 10.1038/srep43261

**Published:** 2017-03-03

**Authors:** Ramona A. J. Zwamborn, Roderick C. Slieker, Petra C. A. Mulder, Inge Zoetemelk, Lars Verschuren, H. Eka D. Suchiman, Karin H. Toet, Simone Droog, P. Eline Slagboom, Teake Kooistra, Robert Kleemann, Bastiaan T. Heijmans

**Affiliations:** 1 Molecular Epidemiology section, Leiden University Medical Center, The Netherlands; 2 Department of Metabolic Health Research, TNO, Leiden, The Netherlands; 3 Department of Vascular Surgery, Leiden University Medical Center, The Netherlands; 4 Department of Microbiology & Systems Biology, TNO, Zeist, The Netherlands

## Abstract

High-fat diets (HFD) are thought to contribute to the development of metabolism-related diseases. The long-term impact of HFD may be mediated by epigenetic mechanisms, and indeed, HFD has been reported to induce DNA methylation changes in white adipose tissue (WAT) near metabolism related genes. However, previous studies were limited to a single WAT depot, a single time-point and primarily examined the pre-pubertal period. To define dynamic DNA methylation patterns specific for WAT depots, we investigated DNA methylation of *Pparg2* and *Leptin* in gonadal adipose tissue (GAT) and subcutaneous adipose tissue (SAT), at baseline and after 6, 12 and 24 weeks of HFD exposure in adult mice. HFD induced hypermethylation of both the *Leptin* promoter (max. 19.6% at week 24, *P* = 2.6·10^−3^) and the *Pparg2* promoter in GAT (max. 10.5% at week 12, *P* = 0.001). The differential methylation was independent of immune cell infiltration upon HFD exposure. In contrast, no differential methylation in the *Pparg2* and *Leptin* promoter was observed in SAT. *Leptin* and *Pparg2* DNA methylation were correlated with gene expression in GAT. Our study shows that prolonged exposure to HFD in adulthood is associated with a gradually increasing DNA methylation level at the *Leptin* and *Pparg2* promoters in a depot-specific manner.

Obesity induced by exposure to a high content of saturated fat diet (HFD) is characterized by hypertrophy and hyperplasia of adipocytes in white adipose tissue (WAT). It is followed by a chronic state of mild inflammation and changes in adipokine secretion, a phenomenon particularly apparent in metabolically active visceral WAT depots, including gonadal adipose tissue (GAT), rather than subcutaneous adipose tissue (SAT)^1^,^2^. Epigenetic changes, such as at the level of DNA methylation, are suggested to contribute to long-term changes in adipokine secretion^2^,^3^,^4^,^5^. In particular, HFD exposure has been consistently linked to differences in DNA methylation near the genes *Leptin* and *Pparg2* (the adipocyte-specific isoform 2 of *Pparg*)^6^,^7^,^8^. The adipokine *Leptin* is a critical signalling component regulating food intake, energy homeostasis, and exhibits potent immunomodulatory functions^9^,^10^. The role of Leptin in satiety signaling is thought be related to an activation of PI3 kinase in the hypothalamus^11^. While in adipose tissue, leptin influences insulin responsiveness via suppressor of cytokine signalling 3 expression which inhibits auto-phosphorylation of the insulin receptor and down-regulates Leptin responsiveness^12^,^13^. In addition to its role in insulin signalling, Leptin has been shown to strongly inhibit lipid synthesis in epididymal adipocytes of normal lean Zucker diabetic fatty rats^14^. *Pparg2* is a master regulator of adipogenesis and is involved in adipocyte differentiation and maturation as well as fat storage and glucose metabolism. Its activation it thought to have insulin sensitizing effects^6^,^15^,^16^. Many of the genes activated by Pparg2 stimulate lipid uptake by adipocytes and adipogenesis or are involved in glucose homeostasis via glucose transporter type 4 (Glut4) and c-Cbl–associated protein (CAP) in adipocytes^15^. Moreover, PPARg2 can control the expression of numerous adipokines such as leptin and tumor necrosis factor-α (TNF-α) in adipose tissue, which can reduce insulin sensitivity^17^.

Until now, studies investigating promoter DNA methylation of *Leptin* and *Pparg2* have primarily focused on HFD exposure *in utero* or in early life^18^,^19^, while few studies examined the effect of HFD in adulthood, a period which may be particularly relevant to the development of obesity in humans (Table 1). Furthermore, insight into the dynamics of DNA methylation differences over prolonged HFD exposure remains scarce and studies did not address the possibility that DNA methylation differences could be confounded by infiltration of immune cells in WAT after HFD exposure. Importantly, the potential difference in response across WAT depots has rarely been explored, although striking differences in morphology and function between depots have been established^20^,^21^,^22^,^23^. In particular SAT and GAT have been implicated in HFD induced obesity^6^,^20^,^21^,^23^,^24^,^25^,^26^. GAT and other abdominal fat depots drive the development of obesity-associated metabolic disorders^25^,^26^, while SAT is considered to be a ‘safe’ storage depot for excess energy without these detrimental effects. Consistent with this view, transplantation of SAT in diet induced obese mice attenuates metabolic dysregulation while its removal exacerbates the condition^27^. Furthermore, GAT is much more prone to inflammation than SAT and its surgical removal attenuates the development of metabolic liver disease in HFD-treated male C57BL/6J mice^26^.

Here, we report on dynamic changes in DNA methylation of the *Pparg2* and *Leptin* promoters during prolonged HFD exposure (at baseline and 6, 12 and 24-week of HFD exposure) in adult male C57BL/6J mice in two different WAT depots, GAT and SAT. In addition, we analysed the impact of immune cell infiltration on DNA methylation and investigated the association between DNA methylation and expression of *Leptin* and *Pparg2*.

## Results

### HFD and body and fat depot weight

We investigated the effect of HFD on bodyweight and mass of GAT and SAT depots as compared with chow after 0 (baseline), 6, 12, or 24 weeks of exposure (n = 11/12 per group). Food intake in HFD and chow groups was isocaloric. Body weight increased with 34.3% after 24 weeks of exposure (*P* < 2.2·10^−16^; Supplementary Table S1). Concomitantly, GAT showed a steep increase in mass during the initial phase of weight gain, reaching its maximum at 12 weeks of exposure (t12: 88.8%, *P* = 4.4·10^−12^), while SAT mass increased more gradually up to 89% after 24 weeks of HFD exposure (*P* = 3.2·10^−11^). The chow control group showed minor and non-significant increases over time confirming that the changes observed were due to the HFD exposure and not an age-related effect (Supplementary Table S1).

### *Pparg2* and *Leptin* methylation during HFD

**Figure 1:**
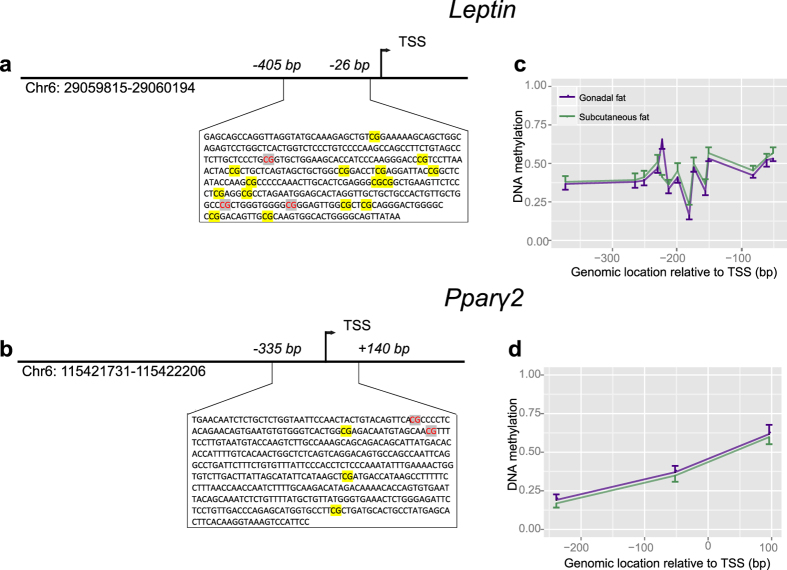
Average DNA methylation of the promoter region of *Leptin* and *Pparg2* before HFD exposure. (**a,b**) Schematic overview of the measured *Leptin* (**a**) and *Pparg2* (**b**) location using reference genome (GRCm38/mm10) and amplicon size; CpGs displayed in red could not be analysed (**c,d**) Average DNA methylation of the *Leptin* and *Pparg2* promoter (y-axis) in each depot for each CpG site relative to the transcription start site (x-axis). Columns represent the mean ± SEM.

**Figure 2:**
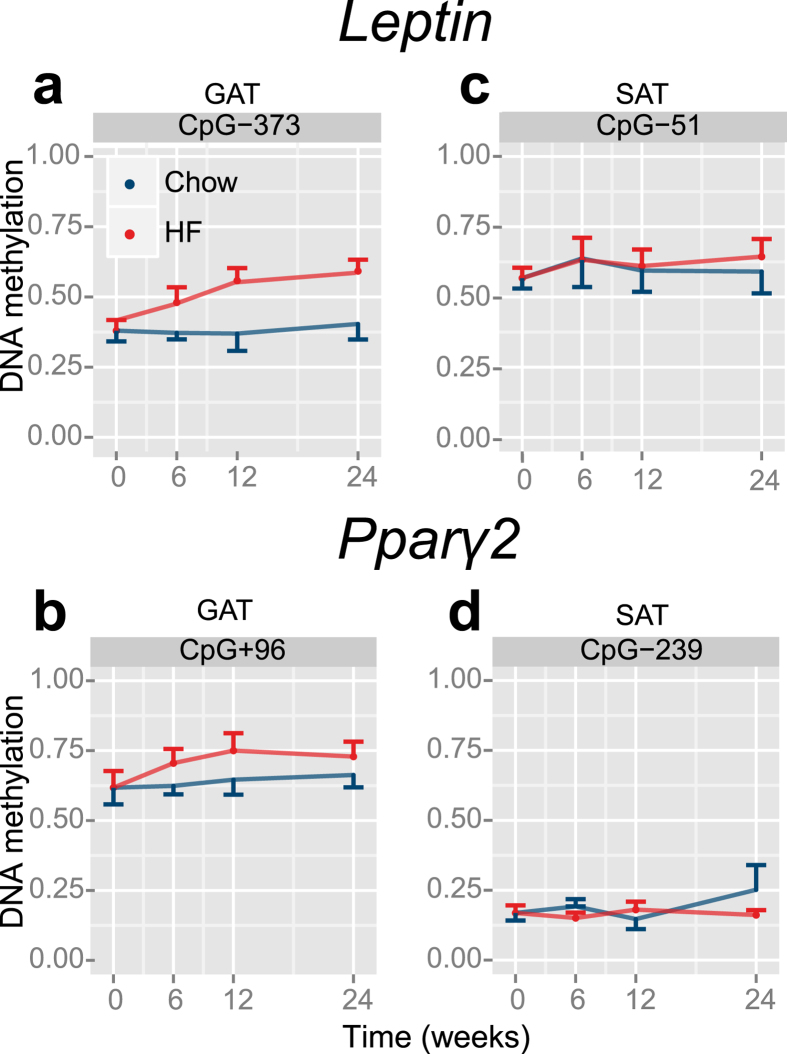
Average DNA methylation after HFD exposure over time. *Leptin* (**a,c**) and *Pparg2* (**b,d**) promoter DNA methylation pattern over time observed in GAT (**a**,**b**) and SAT (**c**,**d**). Line represent the mean ± SEM.

At baseline, DNA methylation levels of the promoters of *Pparg2* and *Leptin* (Fig. 1a,b) were similar in GAT (*P* = 0.68, Fig. 1c) and SAT (*P* = 0.93, Fig. 1d). Over time differences in DNA methylation between the two fat depots were observed in mice exposed to HFD. HFD exposure was associated with increased *Leptin* DNA methylation in GAT (*P*_*HFD*_ = 2.69·10^−25^) over time (*P*_*dynamic*_ = 8.46·10^−3^). Analysis of the individual CpG sites (Supplementary Fig. S1) revealed a maximum increase in DNA methylation of 20% compared to control mice fed a chow diet at the CpG site 373 base pairs downstream of the transcription start site after 24 weeks of HFD exposure (CpG-373, *P* = 7.76·10^−10^, Fig. 2a). Hence, whereas the weight of the GAT reached its maximum at 12 weeks, *Leptin* DNA methylation did at week 24. In SAT, no difference in DNA methylation of the *Leptin* promoter in response to HFD was found (*P* = 0.82, Fig. 2c and Supplementary Fig. S2).

DNA methylation of the *Pparg2* promoter region also increased after HFD exposure (*P* = 8.91·10^4^). In-depth analyses of individual CpG sites in GAT revealed a hypermethylated state of 2 out of 3 measured CpG sites (Supplementary Fig. S3), where CpG + 96 showed the greatest increase in DNA methylation after 12 weeks of exposure compared to the control group (10%, *P* = 6.56·10^−4^, Fig. 2b). Again, no differential DNA methylation was observed in SAT (*P* = 0.46; Fig. 2d, Supplementary Fig. S4). A formal statistical interaction test confirmed the depot-specific DNA methylation due to HFD exposure (*Leptin*: P = 3.65·10^−68^, *Pparg2: P* = 7.04·10^−4^).

### DNA methylation changes are not driven by the infiltration of immune cells

**Figure 3:**
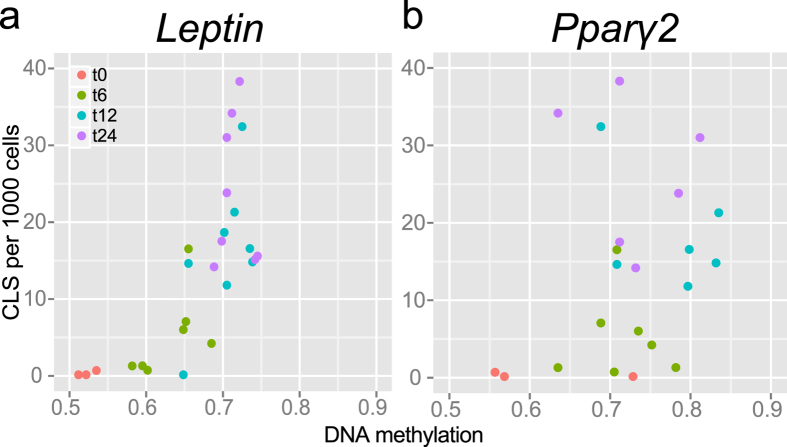
CLS formation over time and relationship with average DNA methylation. (**a,b**) Average DNA methylation correlated with CLS formation per 1000 cells. Individual colours depict different time points.

The increasing storage of lipids in adipose tissue is associated with an increased number of crown-like structures, which are thought to constitute of dead adipocytes that are surrounded by macrophages^21^. This change in cell type composition in the adipose tissue may confound the relationship between HFD and DNA methylation, as the macrophages may have differential DNA methylation compared to adipose tissue. To exclude this confounding effect, we quantified the number of CLS in GAT for a subset of the mice (n = 24). A steep increase in CLS formation was observed after 12 weeks of HFD exposure, with a maximum of 39 CLS per 1000 cells (3.9%) after 24 weeks of exposure to the diet. Next, we compared the number of CLS to the average DNA methylation over time. It was noted that the association between CLS formation and DNA methylation varied profoundly for distinct methylation states in both the *Pparg2* and *Leptin* promoter region (Fig. 3a,b). Subsequently, the effect of CLS formation on DNA methylation was investigated by correcting for CLS formation in a linear mixed model (see statistical analysis). This analysis revealed that CLS formation did not have a significant effect on the DNA methylation (*Leptin; P* = 0.2, *Pparg2; P* = 0.4). Moreover, the effect of HFD exposure on DNA methylation remained significant after correction for CLS in both genes (*Leptin; P* = 0.004, *Pparg2; P* = 0.02). Together, these results indicate that CLS formation did not drive the changes in DNA methylation associated with exposure to a HFD.

### Correlation in *Leptin* and *Pparg2* promoter DNA methylation and gene expression

**Figure 4:**
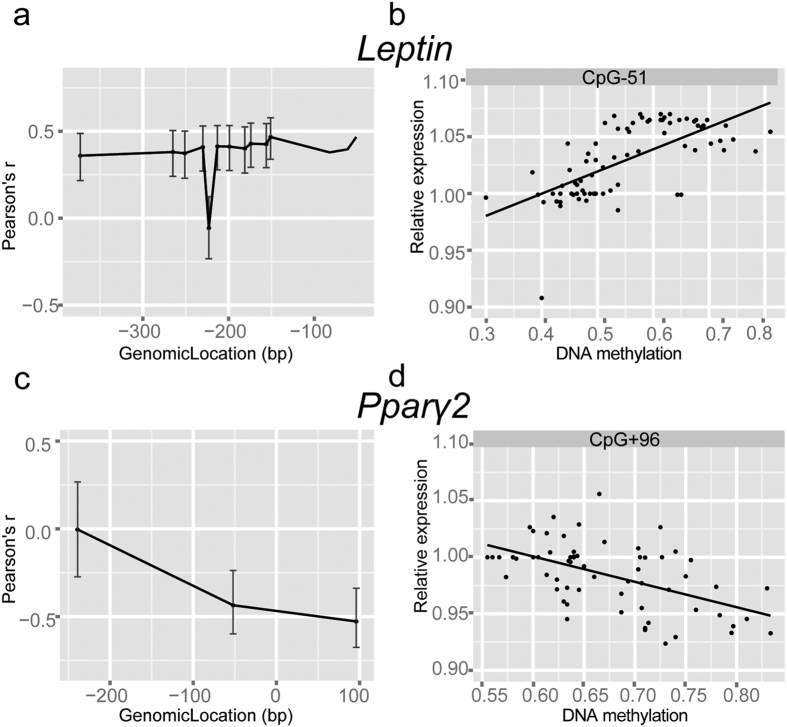
Comparison between *Leptin* and *Pparγ2* gene expression patterns and DNA methylation. (**a,c**) Pearson correlation coefficient between the log expression fold change and individual CpG DNA methylation in GAT and SAT. Columns represent the mean ± SEM (**b,d**) Correlation analysis of DNA methylation of an individual CpG site of *Leptin* and *Pparg2* against the log fold change gene expression.

**Figure 5:**
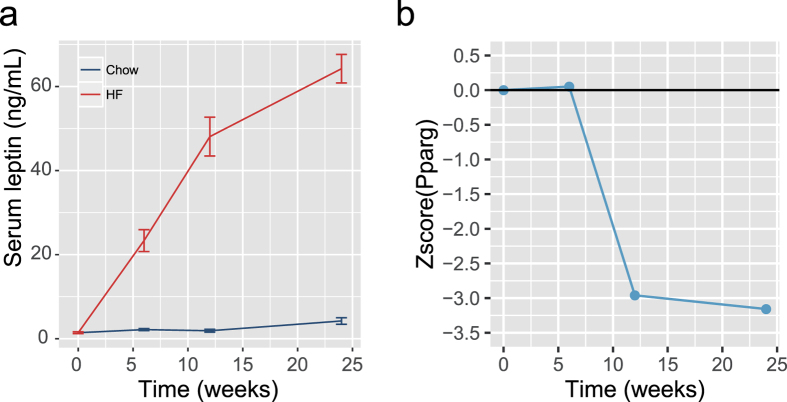
Functional changes for *Leptin* and *Pparg2.* (**a**) *Leptin* plasma levels over time; columns represent the mean ± SEM (**b**) Gene expression changes of Pparg2 target genes over time.

To study the functional consequences of the observed DNA methylation differences of *Pparg2* and *Leptin*, we compared the observed DNA methylation to gene expression of *Pparg2* and *Leptin* in GAT. *Leptin* promoter DNA methylation was positively correlated with gene expression of *Leptin* (Fig. 4a). The correlation with gene expression varied per individual CpG site, with the strongest correlation observed at CpG-51 (r = 0.47, *P* = 2.0·10^−10^, Fig. 4b). The positive correlation obtained from microarray data was confirmed by real-time RT-qPCR for all CpG sites (Supplementary Fig. S5). Furthermore, plasma leptin levels increased over time indicating that the observed effects on DNA methylation and *Leptin* gene expression resulted in an effect on protein level (Fig. 5a).

Analysis of *Pparg2* promoter revealed a negative correlation between DNA methylation and gene expression (Fig. 4c). Again, it was observed that the correlation varied for individual CpG sites, with the strongest correlation observed in CpG + 96 (r = −0.53 *P* = 1.1·10^−6^, Fig. 4d). Although the lack of a *Pparg2*-specific assay precluded direct validation of this result using qPCR, the expression of Pparg2 target-genes were significantly reduced at 12 and 24 weeks of HFD feeding in GAT in line with a lower *Pparg2* expression (Fig. 5b).

## Discussion

We studied the longitudinal effects of long term HFD exposure on DNA methylation of the *Leptin* and *Pparg2* promoters in two fat depots, GAT and SAT, in adult mice during the development of obesity. We found consistently accumulating DNA methylation changes in the promoter regions of the *Leptin* and *Pparg2* genes in GAT over a period of prolonged HFD exposure, whereas DNA methylation remained unaffected in WAT.

In GAT, an increase of 6% was found in *Leptin* promoter DNA methylation starting after 6 weeks of HFD exposure, which reached a maximum increase of 20% after 24 weeks of HFD exposure. Hypermethylation in the *Leptin* promoter after long-term HFD exposure observed is consistent with previous studies in rats^6^ and mice^30^,^31^ after long-term high caloric diet treatment (about 15% increase in DNA methylation). However, discrepancies in the dynamics were observed as some mice studies reported an initial decrease in *Leptin* promoter DNA methylation, after which the DNA methylation increased gradually during high caloric diet exposure^28^,^29^. Different dynamics may be due to the different ages at the start of high caloric diet treatment (e.g. 4–5 weeks of age^31^ versus 12 weeks in this study) and differences in energy density and composition of the diets used (e.g. cafeteria diet in^17^, a high fat diet with a supra-physiological fat content of 60% kcal^31^ versus a more physiological diet we used with a fat content of 45% kcal as is comparable to human diets in Finland and Crete^30^).

At baseline, DNA methylation was similar between GAT and SAT. After HFD exposure, however, an increase in promoter methylation for both the *Pparg2* and the *Leptin* promoter was found in GAT, but not SAT. The difference in DNA methylation between controls and HFD exposed animals increased over time, but remained relatively stable from 12 weeks onwards. Our results show that HFD induces increased *Pparg2* and the *Leptin* promoter DNA methylation in a depot specific manner. DNA methylation changes were observed in metabolically active GAT but not in SAT.

The HFD-induced changes in DNA methylation specifically in GAT indicate differential regulation of the *Leptin* and *Pparg2* genes across depots. The underlying mechanism and physiological consequences of this phenomenon remain unclear, but may be related to reprogramming of the adipokine secretion profile in a depot specific manner. Adipokines are known to reduce the adverse metabolic effects of saturated adipose tissue^25^.

Both *Leptin* and *Pparg2* genes are known to play a role in negative feedback control upon the reduction of body fat. Adipocytes have the ability to regulate *Leptin* expression, which in turn can suppress food intake and permit energy expenditure^31^. Yet, with increasing obesity, tissues can become insensitive to Leptin which is thought to contribute to the progression of obesity^32^. It is thought that the increase in promoter DNA methylation may counteract the steep increase in *Leptin* gene expression^28^. Although it is known that DNA methylation and gene expression can also be positively correlated^33^, an alternative explanation is that *Leptin* promoter DNA methylation is not sufficient to decrease the *Leptin* expression to normal levels^30^. Analysis of plasma leptin showed that the increased DNA methylation and associated gene expression translate in an parallel increase in circulating leptin, indicating an effect on protein level. Hypermethylation of the *Pparg2* promoter, which has been found to be negatively associated with *Pparg2* expression^7^, is hypothesized to reflect an adaptation to further prevent hyperplasia and hypertrophy in already saturated (maximally expanded) adipose tissue by decreasing *Pparg2* expression^7^,^32^. A decrease in *Pparg2* expression results in the decrease in adipose differentiation, insulin responsiveness, lipid uptake and storage through the induction of target genes^16^. However, it is important to note that the observed changes in DNA methylation are not sufficient to make claims regarding protein functionality or biological effects. An analysis of the changes in expression of Pparg2 target genes indicate that the transcriptional activity of Pparg2 was reduced upon HFD. Future (*in vitro*) studies should be performed that focus on the more acute effects of dietary constituents on DNA methylation and how these effects translate into changed expression of target genes and the functional effects (e.g. effect on protein translation or indirect measures such as insulin sensitivity or adipose differentiation).

The inter-depot differences in DNA methylation we report, indicate differential regulation of the *Pparg2* and *Leptin* gene expression in GAT and SAT. This corresponds with previous literature showing that primarily GAT is responsible for the development of metabolic imbalance^23^,^25^,^34^. The depot specific response is thought to arise due to differences in specific adipocyte characteristics^8^. Differences in SAT and GAT in the adult stages have been extensively reported in literature, showing that visceral fat contains a greater percentage of larger adipocytes, is metabolically more active and contains more metabolism-related receptors^35^,^36^. Furthermore, it has been observed that adipogenic progenitors are more abundant in SAT than WAT leading to increased proliferation in SAT but not WAT in response to a high fat diet^37^. The latter could explain the higher sensitivity, early saturation, metabolic dysfunction and DNA methylation changes found in GAT and not SAT after HFD exposure. The presence of inter-depot differences is supported by recent lineage tracing studies showing that both WAT depots originate from different lineages and therefore develop into functionally specific depots^24^,^38^,^39^,^40^. Furthermore, both depots are thought to develop during two different developmental stages. GAT develops postnatally whereas SAT develops prenatally during embryonic day 14 and 18^24^.

It is well-known that saturated GAT accumulates CLS^20^,^25^, a phenomenon also observed in our study. Because adipose tissue and macrophages are known to have a differential DNA methylation profiles, the infiltration of macrophages could have contributed to the observed differential DNA methylation in GAT^41^,^42^. This potential confounding effect has been ignored in previous studies. Importantly, we could exclude that CLS formation drove our findings, implicating that the differential DNA methylation reflects changes in regulation of the *Leptin* and *Pparg2* genes in adipocytes.

In conclusion, we show that prolonged HFD exposure during adulthood dynamically changes DNA methylation and expression of *Leptin* and *Pparg2* genes in GAT. Our study demonstrates that DNA methylation changes induced by prolonged HFD exposure are fat depot specific and may primarily occur in metabolically active depots that become saturated such as GAT, while DNA methylation in SAT remains unaffected. The depot-specific changes in epigenetic regulation may be a starting-point in unravelling the putatively adverse adipokine profiles produced by saturated fat depots that contribute to obesity-induced metabolic imbalance.

## Materials and Methods

### Animals and experimental model

Tissues and plasma were obtained from a subset of mice of a large time-resolved cohort study in C57BL/6J mice^26^. Briefly, eighty 12-week old C57BL/6J mice (Charles River Laboratories, France) were treated with a high fat diet (HFD) with a high content of saturated fat or a control chow diet as previously reported^26^. All animals were housed in a temperature- and humidity-controlled room with ad libitum access to food. The animals were sacrificed by CO_2_ asphyxiation after 0, 6, 12 and 24 (n = 12) weeks of exposure to the diet. After termination, GAT and SAT tissue were collected and partly fixed in formalin for histological analysis as reported^26^ or snap-frozen in liquid nitrogen and stored at −80 °C until further use, e.g. for DNA methylation and mRNA expression analyses performed herein. The histological characterization of the tissues is reported in ref. 26. All animal experiments were performed in compliance with the European Union Council Directive 2010/63/EU and approved by an independent Animal Care and Use Committee of the Netherlands Organization of Applied Scientific Research (Zeist, The Netherlands).

### DNA methylation analysis

Regions in the promoters of the *Leptin* and *Pparg2* genes to target with region-specific DNA methylation assay were taken from previous experimental studies on dietary exposures in rodents^7^,^43^ (for more detailed information regarding promotor location see Supplementary Methods *Leptin, Pparg2*). Locus-specific DNA methylation was measured using the mass-spectrometry method Epityper (Agena Biosciences^®^, Germany). Primers were designed, assays run and data processed as described previously^44^. In brief, all primers were designed with a T7-promoter tag using^®^ EpiDesigner BETA software (http://www.epidesigner.com/) based on the sequences obtained from UCSC genome browser *mm10* (http://genome.ucsc.edu/). *In silico* mass spectrometry using R package *RSeqMeth* was performed to determine the CpG coverage and base mass of the expected PCR products. Details of the primers are shown in Supplementary Methods PCR. DNA was isolated using a phenol/chloroform extraction protocol. Sodium bisulfite conversion was performed on 500 ng genomic DNA using the EZ-96 DNA methylation kit (Zymo Research^®^, USA). To account for possible batch effects the plate was designed with a similar distribution in HFD and chow exposed samples. The bisulfite treated samples were amplified by performing touchdown PCRs using selected PCR primers containing a T7-promotor tag and a 10-mer tag on the reverse and forward primer, respectively (specific PCR conditions are summarized in Supplementary Methods PCR). *In vitro* transcription and T-cleavage reaction were performed using the hMC assay.

DNA methylation ratios were determined by the ratio of C and G spectral peaks using the mass spectrometry–based method Epityper MassARRAY compact MALDITOF (Agena Biosciences^®^, Germany) and analysed by EpiTYPER software 1.2. All measurements were performed in triplicate and individual CpG measurement with ≤2 success rate or with a standard deviation ≥0.1 were discarded. For statistical analysis the average of the triplicate measurements was used.

### Microarray and RT-PCR analysis

Total RNA was extracted from adipose tissues using glass beads and RNAbee (Tel-Test Inc, Friendswood, USA). RNA integrity was examined using the RNA 6000 Nano Lab-on-a-Chip kit and a Bioanalyzer 2100 (Agilent Technologies^®^, Amstelveen, The Netherlands). The Illumina^®^ TotalPrep™ RNA Amplification Kit (Ambion, art.No.AM-IL1791) was used to synthesize biotin labeled cRNA starting with 500 ng total RNA. The biotinylated cRNA was then hybridized onto the MouseRef-8 Expression BeadChip was 750 ng. Lastly, the default setting of Illumina’s Genomestudio v1.1.1 was used for Gene Expression analysis. All the quality control data of this BeadChip were within specifications of the microarray service provider (Service XS, Leiden, the Netherlands). The microarray gene expression data were validated using quantitative real-time PCR for *Leptin* using established protocols and primer/probe sets^45^. Ingenuity Pathway Analysis (IPA) was used to analyse microarray data and Pparg2 target genes. The upstream regulator analysis tool of IPA was used to determine the transcriptional activity of Pparg2 transcription factor essentially as reported^45^. A negative Z-score <−2 indicated a reduced transcriptional activity based on the direction of gene expression changes of target genes.

### Biochemical analyses

Plasma leptin levels were determined by a Quantikine ELISA for murine leptin (R&D Systems^®^, UK).

### Statistical analyses

First, the effect of HFD exposure on the developed fat mass and bodyweight was analysed using a one-way analysis of variance that accounted for the effect of individual differences in time, HFD exposure and the interaction between time and exposure.

Secondly, exposure and/or time specific differences in DNA methylation after HFD exposure in the whole measured region were analysed. To analyse this effect, linear mixed models were used. The analyses accounted for individual differences between CpG sites (*CpG ID* ∗ *b*_1_), age (*Time* ∗ *b*_3_) and individual variation of the mice (*Mouse ID ∗ b*_*2*_). To test for time specificity, the interaction between time and exposure was added to the model (Equation 1):


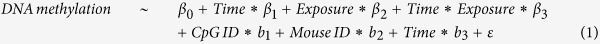


Furthermore, the effect of HFD between tissues was evaluated using a linear mixed model similar to the abovementioned model, however depot DNA methylation was added as a fixed effect and the interaction between Exposure * Depot DNA methylation was investigated instead of the interaction between Exposure*Time.

To calculate the effect in individual CpG sites over time, pairwise t-tests were applied to determine the effects of HFD exposure for every time point. *P*-values were corrected for multiple comparisons following the Bonferroni procedure and considered significant at *P*_*adj*_ ≤ 0.05.

The effect of HFD exposure on expression levels of *Pparg2* and *Leptin* was investigated using a linear mixed model corrected for age (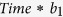) (Equation 2):


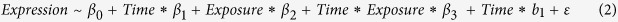


The relationship between gene expression and DNA methylation was calculated based on the Pearson correlation coefficients between gene expression and DNA methylation per CpG and corrected for multiple testing using the Bonferroni procedure.

Finally, to test for the confounding effect of CLS formation on DNA methylation a linear mixed model was applied on the data. The variable CLS (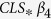) was added to the model as fixed effect and the interaction between time and exposure was removed (Equation 3):


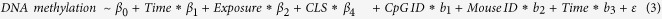


All analyses were performed using R statistics version 3.1.2 (http://www.r-project.org/) with the packages: *ggplot2* and *lme4*.

## Additional Information

**How to cite this article:** Zwamborn, R. A. J. *et al*. Prolonged high-fat diet induces gradual and fat depot-specific DNA methylation changes in adult mice. *Sci. Rep.* **7**, 43261; doi: 10.1038/srep43261 (2017).

**Publisher's note:** Springer Nature remains neutral with regard to jurisdictional claims in published maps and institutional affiliations.

## Supplementary Material

Supplementary Data

## Figures and Tables

**Table 1:** Effects of adult HFD exposure on *Leptin* and *Pparg2* DNA methylation and gene expression reported in literature.

Gene	Study	Species/Strain/Sex	Group size	Tissue	HFD	Time	Start diet	Expression	DNA methylation
*Leptin*	28	Male C57BL/6 J	n = 12 chow/HF	Epididymal fat (GAT)	34.9% fat by wt	4/8/12/18 weeks	4/5 weeks	mRNA *Leptin*↑	*Leptin*↑
	29		n = 15 chow/HF						
*Pparg2*	7		n = 3 chow/HF	Inguinal and Epididymal fat (GAT)	n.a.	16 weeks	4 weeks	mRNA *Pparγ2*↓	*Pparγ2*↑
*Leptin*	6	Wistar rats	n = 5 chow/n = 6 HF	Retroperitoneal fat	59.2% energy from fat	11 weeks	post weaning	n.a.	*Leptin*↑
	46	Wistar rats	n = 12 chow/HF	Retroperitoneal fat	45% fat by wt	20 weeks	3 weeks	n.a.	*Leptin*↑
