# Supplementary material for: Prolonged high-fat diet induces gradual and fat depot-specific DNA methylation changes in adult mice
Source: Sci Rep. 2017 Mar 3;7:43261. doi: 10.1038/srep43261 (PMC5335669; doi:10.1038/srep43261)
Supplement: Supplementary Data [file srep43261-s1.pdf]

Prolonged high-fat diet induces gradual and fat depot-specific DNA methylation changes in adult mice

Ramona Zwamborn[1,], Roderick Slieker[1,*], Petra Mulder[2,3*], Inge Zoetemelk[1], Lars Verschuren[2], Eka Suchiman[1] , Karin Toet[2], Simone Droog[2], Eline Slagboom[1], Teake Kooistra[2], Robert Kleemann[2], Bastiaan Heijmans[1]

SUPPLEMENTARY LEGEND

Supplementary table S1

Overview of bodyweight and fat mass development after exposure to both dietary conditions

		T=0	T= 6		T=12		T=24	
		Mean ± SD	*Mean ± SD*	*P-value*	*Mean ± SD*	*P-value*	*Mean ± SD*	*P-value*
GAT mass	HF	-	1.83 ± 0.62	7.34E-09	2.43 ± 0.46	$4.35*10^{-12}$	1.76 ± 0.35	$3.39*10^{-05}$
	Chow	0.39± 0.04	0.55 ± 0.15		0.52 ± 0.2		0.8 ± 0.37	
SAT mass	HF	-	0.82 ± 0.23	5.59E-10	1.54 ± 0.45	$6.18*10^{-12}$	2.18 ± 0.33	$3.23*10^{-11}$
	Chow	0.24 ± 0.02	0.27 ± 0.05		0.26 ± 0.33		0.37 ± 0.13	
Bodyweight	HF	-	36.21 ± 4.39	1.97E-05	44.25 ± 5.42	$2.81*10^{-07}$	51.25 ± 2.75	$1.80*10^{-07}$
	Chow	26.91 ± 0.88	29.74 ± 1.36		31.8 ± 1.82		33.65 ± 1.77	

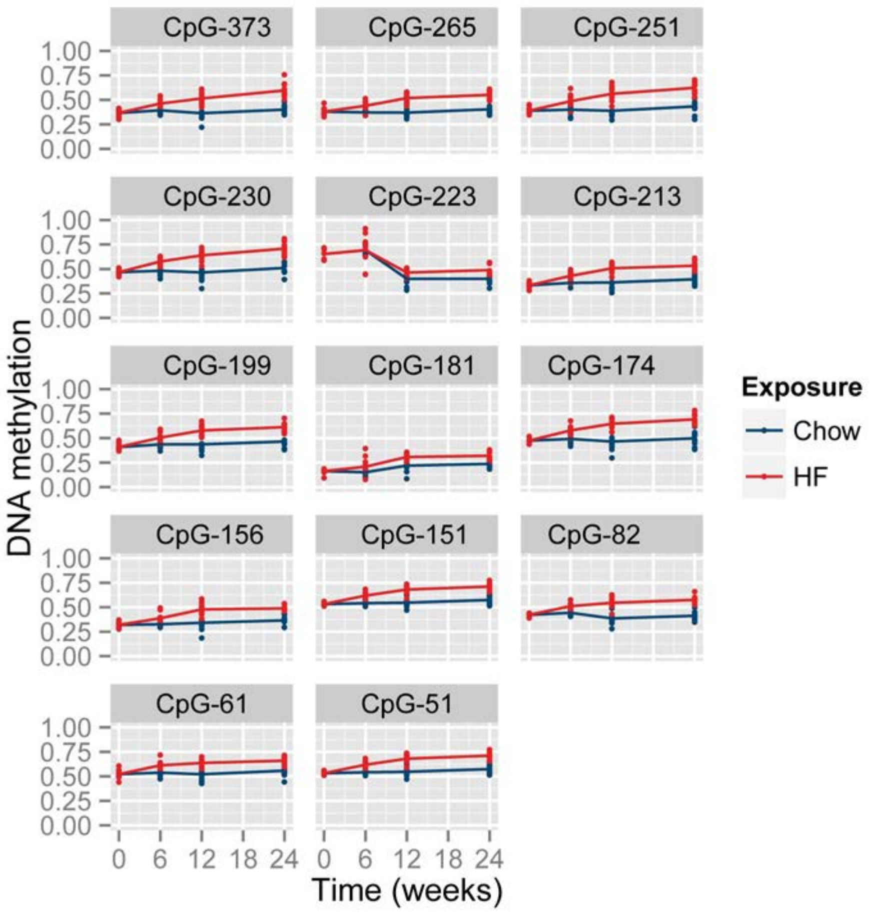

Supplementary Figure S1

Overview of the average DNA methylation of the Leptin promoter region in GAT for every individual CpG site.

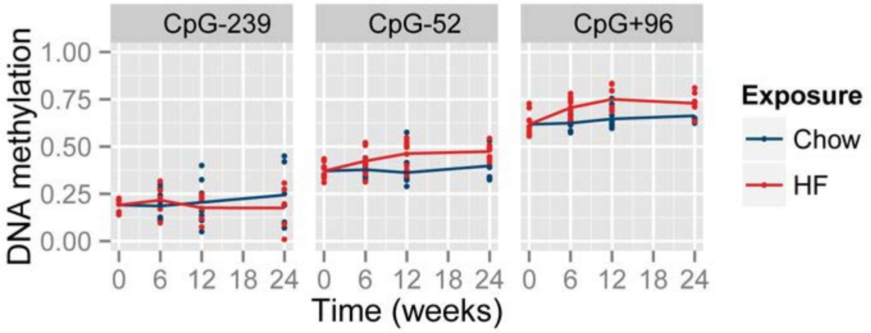

Supplementary Figure S2

Overview of the average methylation of the Pparg2 promoter region in GAT for every individual CpG site.

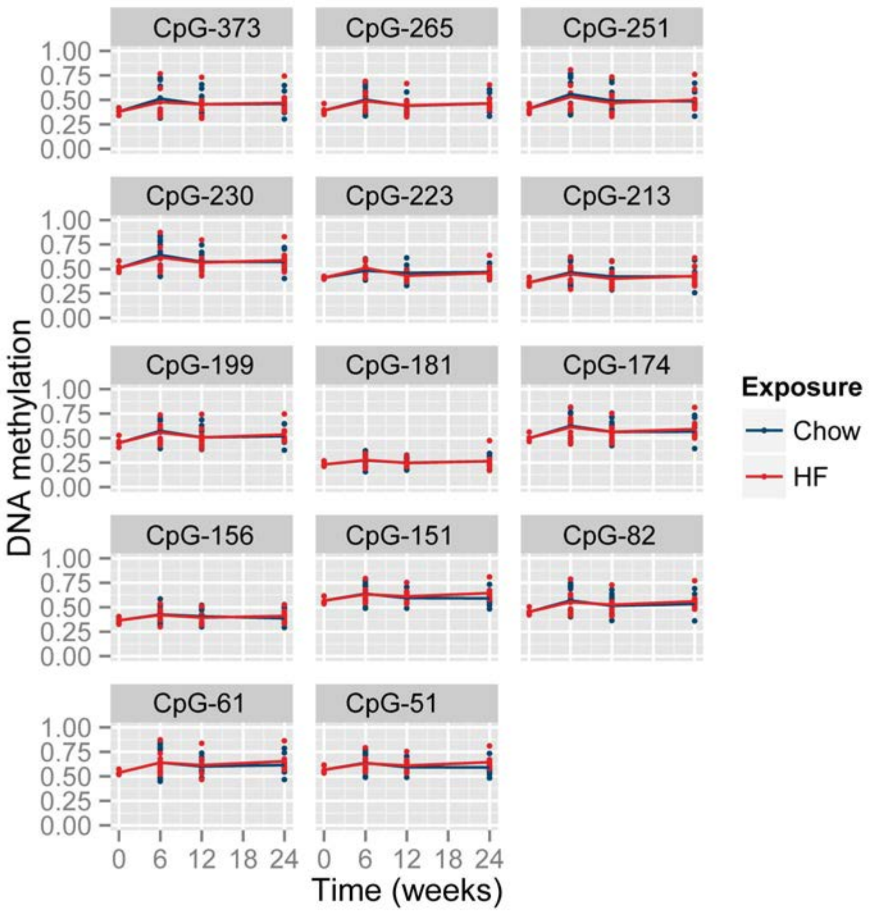

Supplementary Figure S3
Overview of the average DNA methylation of the Leptin promoter region in SAT for every individual CpG site.

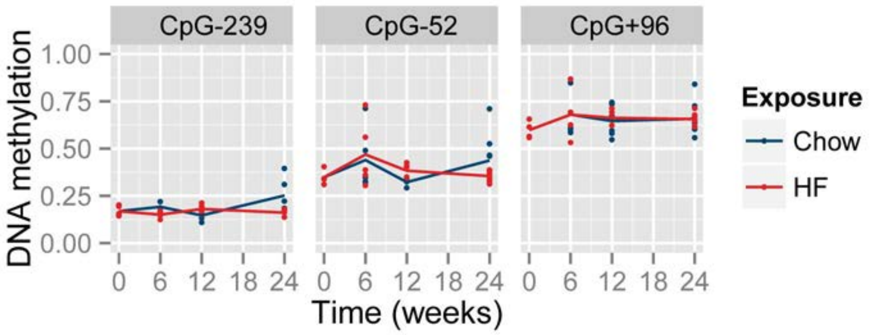

Supplementary Figure S4
Overview of the group averages and statistical analysis of the Pparg2 promoter region in SAT for every individual CpG site.

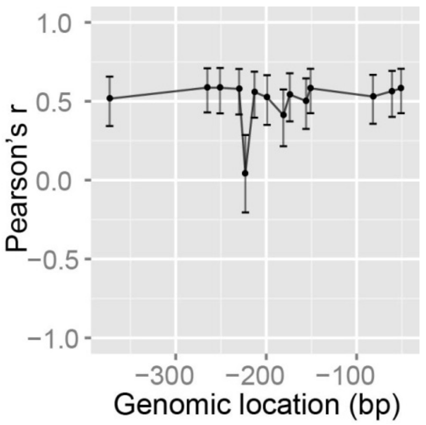

Supplementary Figure S5

Overview of the Pearson correlation coefficients between the log expression fold change and individual CpG DNA methylation for the RT-qPCR

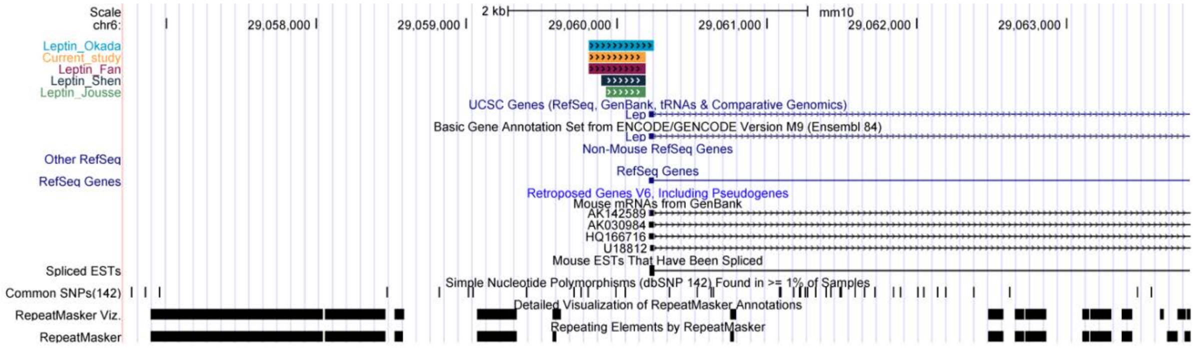

Supplementary Methods ***Leptin***

General promotor Pparg2 location described in previous studies compared to the promotor location of the current study.

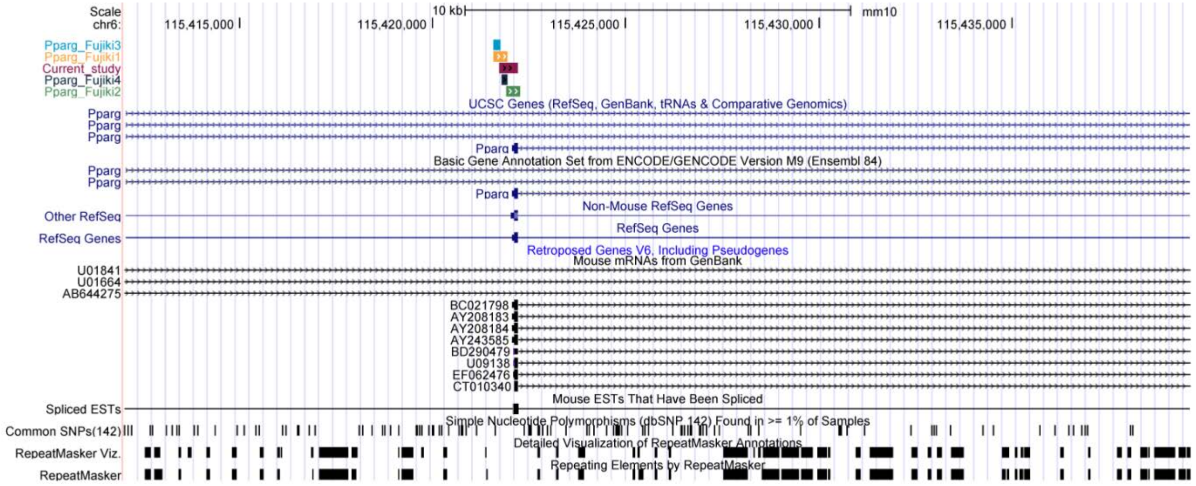

Supplementary Methods ***Pparg2***

General promotor Pparg2 location described in previous studies compared to the promotor location of the current study.

Supplementary Methods PCR

PCR reagents:
The test touchdown PCR procedure was carried out in a reaction volume of 5 µl containing; 1.42 µl MilliQ, 0.5 µl 10x HotStarTaq PCR Buffer (Qiagen, Germany), 0.04 µl dNTPs (25mM) (Sequenom, USA), 0.04 µl HotStarTaq DNA polymerase (Qiagen, Germany), 1 µl primer mix (1 pmol F/R) (Metabion, Germany) and 2 µl bisulphite converted DNA (5 ng/µl).

Primer sequence for in vitro transcription and PCR conditions.
For the PCR on bisulphite-treated genomic DNA, the following PCR conditions were used: 1X 95°C for 15 min; 4X 95°C for 20 s, 65°C for 30 s and 72°C for 60 s; 4X 95°C for 20 s, 58°C for 30 s and 72°C for 60 s; 38X 95°C for 20 s, Ta for 30 s and 72°C for 60 s; 1X 72°C for 3 min.

**Gene**	**Genomic location** *(Build: NCBI37/mm9)*	**Primer**	**Ta (°C)**	**Primer sequence (5' --> 3')**	**Length (bp)**	**Product size/ assayed CGs (bp/n)**
Leptin	Chr6: 29059815 - 29060194	Forward 10F	58	GAGTAGTTAGGTTAGGTATGTAAAGAG	27+10	379/19
		Reverse T7R		TAATAACTACCCCAATACCACTTAC	24+31	
Peroxisome proliferator-activated receptor gamma2 *(Pparγ2)*	Chr6: 115421731 - 115422206	Forward 10F	58	GGAATGGATTTTATTTTGTGAAGTGT	26+10	475/3
		Reverse T7R		AAACAATCTCTACTCTAATAATTCCAAC	27+31	
